# Are Soccer Players Older Now Than Before? Aging Trends and Market Value in the Last Three Decades of the UEFA Champions League

**DOI:** 10.3389/fpsyg.2019.00076

**Published:** 2019-01-28

**Authors:** Anton Kalén, Ezequiel Rey, Alejandro Sal de Rellán-Guerra, Carlos Lago-Peñas

**Affiliations:** Faculty of Education and Sport Sciences, University of Vigo, Pontevedra, Spain

**Keywords:** aging trend, peak age, soccer, performance analysis, market value

## Abstract

The aims of the current study were to analyze the evolution of players’ age in the UEFA Champions League since the start of its modern-day format in 1992–1993 up until 2017–2018 and to determine how the players’ age relates to their market value. The sample consisted of all players participating in the UEFA Champions League from the 1992–1993 to 2017–2018 seasons (*n* = 16062). The following variables were used in this study: players’ age, number of seasons in the club, number of Champions Leagues won, team performance, and market value of the player in the season. Data were examined using a one-way ANOVA and a linear regression. The main finding of the current study is that an aging trend has occurred in the last three decades in the Champions League. A significant increase in average players’ age (>1.6 years) was observed, rising from an age of 24.9 to 26.5 years. Goalkeepers and Center Backs tend to peak later than attackers, and their peak performance can last until an age of about 31 years. Finally, an inverted-U curve defines the association between market value and age, with peak value appearing in the 26–30 age range. These results provide useful information regarding at which age soccer players are likely to perform at the highest level, as well as the age they are likely to have the highest market value.

## Introduction

Professional soccer teams usually consist of players from a wide age range (Dendir, 2016). In the four major European professional soccer leagues, Fußball-Bundesliga (Germany), Premier League (England), Serie A (Italy), and La Liga (Spain), most players are between 21 and 29 years old, and a substantial drop-off is observed at around the age of 29 years (Dendir, 2016). Moreover, there is a general belief that players usually peak somewhere in their mid to late twenties, with attacking players tending to peak earlier than defenders (Kuper, 2011; Caley, 2013). However, this is based mainly on anecdotal evidence and views of professionals in the game and less on scientific research. In a recent study, Dendir (2016) also found that the average professional soccer player in the major leagues in Europe peaks between the ages of 25 and 27, where the average forward peaks at 25 while defenders at 27. For midfielders, the peak age occurs in the 25–27 age range. Also, several top European soccer clubs have adopted an unofficial contract policy with shorter contracts lengths as players are nearing 30 years, based on a belief that elite players are well past their peak performance after this age (Dendir, 2016). Thus, the age of professional soccer players and at which age professional soccer players peak seems to be an important variable of interest not only to performance analysts and sport scientists, but also managers and coaches. The perception of when players tend to peak can affect the soccer club’s personnel decisions, such as the length of contracts offered to players and the acceptable sum of transfer fees when buying or selling players (Dendir, 2016). Knowledge of when players are in their optimal age, therefore, has substantial value for the soccer industry. From a sport perspective, this provide useful information regarding at which age soccer players are likely to perform at the highest level.

However, while the evolution of tactical, technical and physical performance over time have been studied extensively (e.g., Barnes et al., 2014; Wallace and Norton, 2014; Bush et al., 2015), to the best of our knowledge, no studies have examined the aging trend of male elite soccer players in recent decades. Conversely, aging trends in different individual and team sports like baseball (Fair, 2008), tennis (Kovalchik, 2014), or triathlon (Rüst et al., 2012), among others, have been previously investigated, suggesting a marked increase in the age of peak performance of elite athletes during the last two decades, probably due to factors such as advances in sport science and technology (Allen and Hopkins, 2015). In tennis, for example, Kovalchik (2014), p. 8) found that the average age of the top 100 male players has increased over the last decade at a pace of 0.34 years per season since the mid-2000s, rising from the age of 26.2 years to an all-time high of 27.9 years. Given this evidence, there is a clear need to investigate trends in the age of peak performance among top professional soccer players in order to provide important clues about the sport’s evolution and may help to create more specific strategies to increase players’ performances in the future.

Chronological age of highest performance differs among sports (Smith, 2003) and depends on different biological capabilities involved in each sport and by the specific skills and attributes needed to succeed (Allen and Hopkins, 2015). This argues the evidence that physiological and technical constraint of each sport dictates the window for optimal performance (Dendir, 2016). At this respect, peak window of mid-20s estimated by Dendir (2016) seems to be explained by the combination of endurance and explosive power necessary to cope with physical and physiological demands of modern elite soccer. However, even though the aging process influences the players’ physical and mental development and, in turn, their competitive performance (Allen and Hopkins, 2015), there are no scientific studies that have examined the evolution of players’ age in elite soccer.

Taking all previous considerations into account, this study aims to analyze the evolution of players’ age in the UEFA Champions League since the start of its modern-day format in 1992–1993 up until the 2017–2018 season. It also aims to analyze if the players’ age has evolved differently depending on playing position or team level. Lastly, it aims to analyze how the players’ age relates to their market value. We hypothesize that the average age of the Champions League players has increased across all positions and team levels. We further hypothesize that an inverted-U curve defines the association between market value and age, with peak value occurring at the mid-20s.

## Materials and Methods

### Sample

The sample consisted of all players participating in the UEFA Champions League from the 1992–1993 to 2017–2018 seasons that played at least in one match (*n* = 16062). Each participation of a player in a season was recorded as an individual case, i.e., a single player can represent multiple cases. The players were classified into six positions: Goalkeepers (GK, *n* = 1224), Center Backs (CB, *n* = 3206), Fullbacks (FB, *n* = 2383), Center Midfielders (CM, *n* = 4609), Wingers (W, *n* = 1980), and Forward (F, *n* = 2660). This classification was done according to the information provided by the official UEFA website ^1^.

### Variables

The following variables were used in this study: players’ age, number of seasons in the club, number of Champions Leagues won, team performance, and market value of the player in the season. Players’ age was calculated as the date of competition minus the date of birth according to the information provided by the official UEFA website (see footnote 1). In line with previous studies and for posterior analyses (Botek et al., 2016), soccer players were divided into five age groups: 16–20, 20–25, 26–30, 30–35 and >35 years. The team performance was decided by how far the team reached in the Champions League: Winners, Final, Semifinal, Quarterfinal, Round of 16 and Group Phase. The number of seasons in the club and number of Champions Leagues won by each player was obtained from the UEFA’s website (see footnote 1). Finally, according to previous research and due to the difficulty of operationalizing performance in a mixed/skill-based sport like soccer, the market value of the player in the season (Gerhards and Mutz, 2017) was obtained from the Transfermarkt website ^2^.

### Statistical Analysis

Statistical analyses were conducted using IBM^®^ SPSS^®^ Statistics 21 for Macintosh (IBM Co., New York, NY, United States), except for regression analyses that were conducted using STATA (version 15.1, TX, United States). Results are reported as means and standard deviations (mean ± SD). Statistical significance was set at *p* < 0.05. The assumption of normality of the data was checked both graphically and using the Kolmogorov–Smirnov test. All data were normally distributed. The homogeneity of variances was examined using Levene’s test. As the samples were normally distributed and displayed homogenous variance, a one-way analysis of variance (ANOVA) was used to evaluate differences in mean ages across different playing positions. Subsequently, one-way independent-measures ANOVA test with sphericity assumed was used to compare mean age from each season. In the event of a difference being present, Bonferroni-adjusted *post hoc* tests were used to identify specific effects.

Moreover, the effects of the age of the players (AGE), the playing position (PP), the number of seasons in the club (NS) and the number of Champions Leagues won (NCL) on the market values of the players were also examined through a linear regression model. Positive or negative coefficients indicate a greater or lesser market value of the players, respectively. β_1_ is the intercept; β_2_, β_3_, β_4_, and β_5_ are the impacts of each of the independent variables; and 𝜀_1_ is the error term. The model is as follows:

$$\text{Market}\;\text{Value}={\text{β}}_{1}+{\text{ β}}_{2}\;\text{AGE}+{\text{β}}_{3}\text{PP}+{\;\beta }_{4}\text{NS}+{\;\beta }_{5}\text{NCL}+{\text{ ε}}_{1}$$

## Results

**FIGURE 1:**
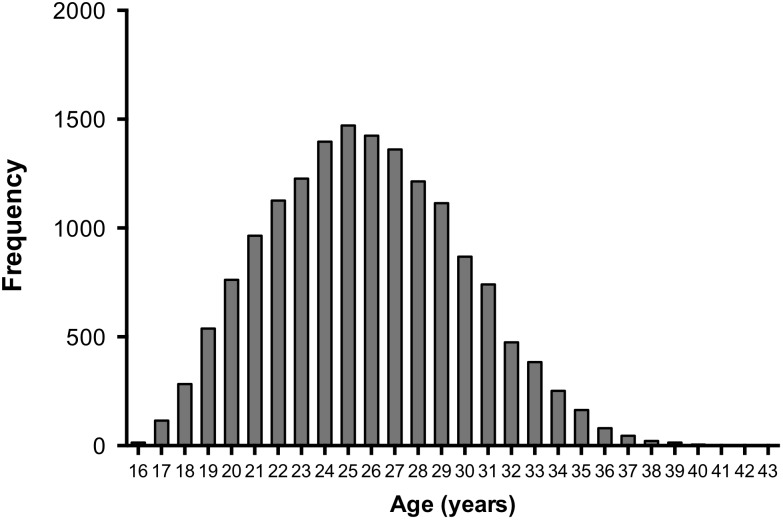
Age distribution of UEFA Champions League players from 1992–1993 to 2017–2018.

**FIGURE 2:**
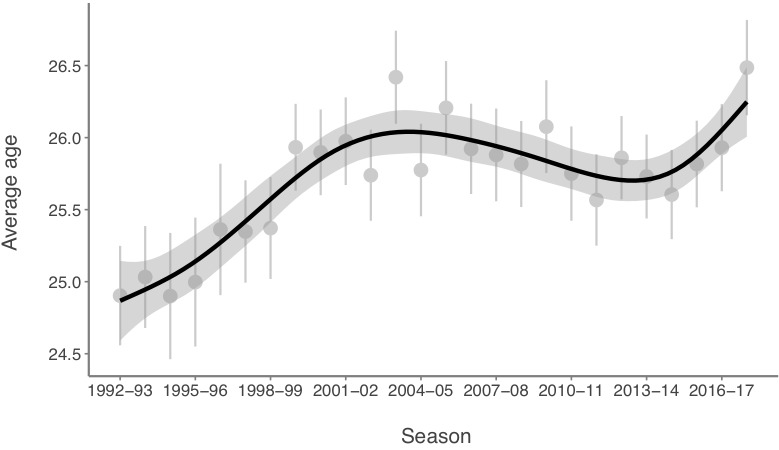
Age trend of UEFA Champions League players.

The histogram of the age distribution for players included in the study is presented in Figure 1. The age of the players ranges from 16 to 43, with an average of 25.75 ± 4.14 years. A large number of players were observed between 21 and 29 years (>80%). From 29 years an onward, there is a substantial yearly decrease in the number of players. Between the 1992–1993 and 2017–2018 seasons, a significant increase in the players’ average age (>1.6 years) was observed (*p* < 0.001). However, this increase was not uniform, and two break-points were identified along these seasons, the first one in season 2003–2004 and the second one in season 2013–2014, which can be observed in Figure 2.

**FIGURE 3:**
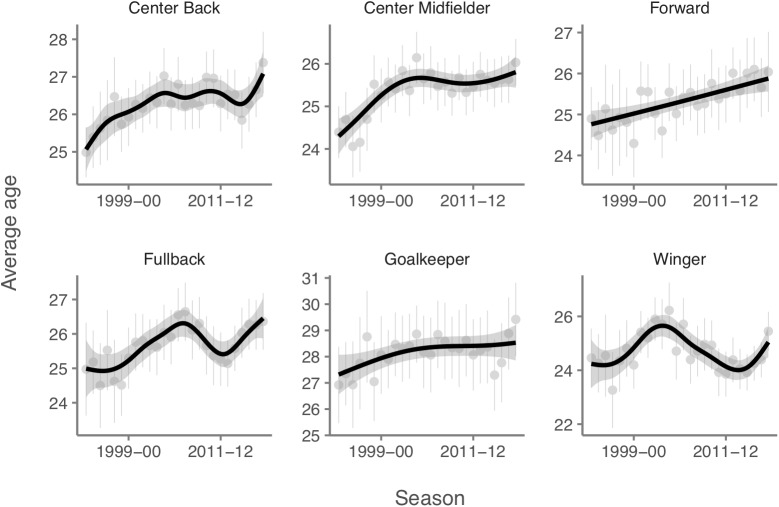
Age trend by playing position.

With all seasons pooled, the one-way ANOVA showed significant differences between positional roles on mean age (*p* < 0.001). GK (28.19 ± 4.66 years) and CB (26.31 ± 4.13 years) showed significantly higher mean ages than F (25.32 ± 3.92 years), W (24.70 ± 3.90 years) and CM (25.44 ± 3.99 years). Although an aging tendency was apparent for all playing positions between the 1992–1993 and 2017–2018 seasons, a more stable trend was observed in F, CM, and GK compared to CB, W, and FB (Figure 3).

**FIGURE 4:**
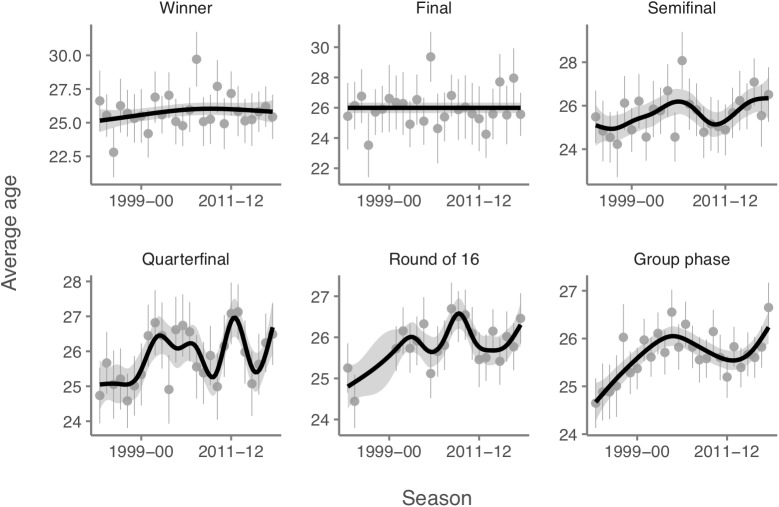
Age trend by team performance.

As can be seen in Figure 4, although an aging trend was found for all categories of team performance considered, no significant differences were found between winners, finalists or semifinalists and the other categories.

Effects of independent variables on the market values of the players are displayed in Table 1. Players 21–25 and 26–30 years old have a higher market value (*p* < 0.01) compared to players 16–20 years old (reference category in the regression model). However, players 31–35 and more than 35 years old have a lower market value than players 16–20 years old (*p* < 0.01). Concerning the playing position, F, W, and CM are more expensive than GK (reference category in the regression model), while no differences were found between CB, FB and GK.

**Table 1:** The influence of the age of the players, playing position, number of seasons in the club and number of Champions Leagues won on the market values of soccer players.

Variables	Coefficients
**Age**	
21–25 years	4,361,629 (442,206)^∗^
26–30 years	5,024,691 (449,663)^∗^
31–35 years	–2,300,026 (550,657)^∗^
>35 years	–8,790,432 (1,263,247)^∗^
**Playing Position**	
Center Back	524,204 (543,055)
Fullback	–703,459 (562,786)
Central Midfielder	2,238,671 (521,619)^∗^
Winger	3,979,548 (580,273)^∗^
Forward	4,931,916 (567,981)^∗^
Seasons in the Club	435,123 (47,849)^∗^
Champions Leagues won	8,238,528 (620,846)^∗^
Intercept	1,808,392^∗^
*R*^2^	0.18
Number of observations	8665


*^∗^p < 0.01*

The more seasons a player stays in a club, the higher their market value is. For each season in the club, the value of the player increases by €435,123 (*p* < 0.01). Finally, the number of Champions Leagues won by the players has a significant effect on their market value; each Champions League won increases their market value by more than €8 million.

## Discussion

This study provides new information about the evolution of professional soccer players’ age of peak performance. The principal finding of the present study is that an aging trend has occurred in the last three decades in the UEFA Champions League. Previous studies (Kuper, 2011; Caley, 2013; Dendir, 2016) have demonstrated that professional soccer players peak around their mid-20s. However, none of these studies have analyzed the aging pattern in elite soccer. It seems that the evolution of contemporary soccer is probably associated with increasing age of athletes. Several factors may have contributed to recent “aging” of the top players. One of the factors is likely the increased investments of football clubs on support functions to monitor, increase and sustain the players’ performance; including modern training facilities, strength and conditioning departments and dietists (Anderson and Sally, 2013; Williams, 2013; Dendir, 2016).

Although an aging tendency has occurred for all playing positions between the 1992–1993 and 2017–2018 seasons, GK and CB tend to peak later than F. Recently, Dendir (2016) found that the average forward and defender peaks at 25 and 27, respectively. For midfielders, the peak age occurs in the 25–27 age range. These findings can be largely explained by differences in the physical demands of playing each position, which has previously been heavily studied (Bangsbo et al., 1991; Rienzi et al., 2000; Mohr et al., 2003, 2008; Bradley et al., 2009, 2010, 2011; Di Salvo et al., 2009). Using time-motion analysis, these studies have shown that forward performs both higher number of (and longer) maximal sprints, higher number of shuffles, more contact at high intensity and higher amount of high and very high intensity activities; defenders the spend the least time running and sprinting, while midfielders the most (Bangsbo et al., 1991; Mohr et al., 2003, 2008; Bradley et al., 2009, 2010, 2011; Di Salvo et al., 2009). The lower physical demand for defenders is likely one of the reasons they tend to peak at a later age, as well as maintaining a high performance higher up in age. In similar fashion, the higher amount of high-intensity activity is probably one of the causes of the earlier peak of forward.

Conventional wisdom suggests that there is a perfect age to be a successful player. The average age of the 32 teams that participated in the last two World Cups was 27.5 and 27.37. It has been found that a one-year increase in average team age results in a performance drop of four positions in the World Cup (Dendir, 2016). According to our results, although an aging trend was found for all team performance categories considered, no significant differences were found between winners, finalists or semifinalists and the other classifications. These results may be due to the fact that players from all over the world participate in the Champions League, and the differences between the participating teams probably are smaller than in the World Cup. Future studies should provide more information about the relationship between aging trend and success in elite soccer.

The results confirm the hypothesis that an inverted-U curve characterizes the relationship between market valuation and the age, with peak value occurring in the 26–30 age range. These results are similar to those provided in other studies. For example, Anderson and Sally (2013) found that the peak value in the Premier League occurs at age 26. There is a substantial drop-off in the market value at the 31–35 age range. Finally, players over 35 have the lowest market value. In regard to playing position, attackers have a higher market value than defenders. That is, the closer the opponent penalty area, the higher the market value of the players, with forward having the highest market value.

A limitation in the current study is that extraneous variables that might have some effects the results where not included (Gómez et al., 2013). For example, the own and opponent team’s formation might affect the amount of players the teams contract for each position. The fact that anonymized data was used in the study mean that some observation might correspond to the same player. Finally, it has not been controlled that the players occupied the same playing position or that they have played in more than one team during the analyzed seasons.

In conclusion, the results in the paper confirm that (i) an aging trend has occurred in the last three decades in the Champions League, and that (ii) GK and CB tend to peak later than attackers, and their peak performance can be maintained longer, until an age of about 31 years. The current results provide useful information in terms of informing our expectations about when soccer players are likely to perform at the maximum level. They also inform us about when they are likely to be the most valuable in the market. From a player’s recruitment or renovation perspective, the current findings can provide valuable information to assist in decisions regarding recruitment and player list management within professional soccer clubs. When a new contract will be signed, the duration of the contract or the salary of the players can now be decided more objectively. Similarly, post-match assessment of the technical, tactical and physical aspects of performance can be made more objectively by factoring in the effect of the age of the players and can help managers and coaches to guide decisions regarding individualizing training strategies or design training load periods.

## Author Contributions

CL-P and ER conceptualized the study. AR-G performed the data curation. CL-P, ER, and AK involved the formal analysis. CL-P, ER, AK, and AR-G designed the methodology. CL-P administrated the project. CL-P supervised the study. AK visualized the study. CL-P, ER, and AK wrote the manuscript.

## Conflict of Interest Statement

The authors declare that the research was conducted in the absence of any commercial or financial relationships that could be construed as a potential conflict of interest.

## Footnotes

1 www.uefa.com

2 www.transfermarkt.com
